# Broadscale spatial synchrony in a West Nile virus mosquito vector across multiple timescales

**DOI:** 10.1038/s41598-024-62384-6

**Published:** 2024-05-30

**Authors:** Lindsay P. Campbell, Amely M. Bauer, Yasmin Tavares, Robert P. Guralnick, Daniel Reuman

**Affiliations:** 1 Florida Medical Entomology Laboratory, University of Florida, Vero Beach, FL 32962 USA; 2 Department of Entomology and Nematology, University of Florida, Gainesville, FL 32611 USA; 3 Department of Ecology, Evolution, and Environmental Biology, Graduate School of Arts and Sciences, Columbia University, New York, NY 10025 USA; 4 Florida Museum, University of Florida, Gainesville, FL 32611 USA; 5 Department of Ecology and Evolutionary Biology and Center for Ecological Research, University of Kansas, Lawrence, KS 66047 USA

**Keywords:** Ecology, Population dynamics

## Abstract

Insects often exhibit irruptive population dynamics determined by environmental conditions. We examine if populations of the *Culex tarsalis* mosquito, a West Nile virus (WNV) vector, fluctuate synchronously over broad spatial extents and multiple timescales and whether climate drives synchrony in *Cx. tarsalis*, especially at annual timescales, due to the synchronous influence of temperature, precipitation, and/or humidity. We leveraged mosquito collections across 9 National Ecological Observatory Network (NEON) sites distributed in the interior West and Great Plains region USA over a 45-month period, and associated gridMET climate data. We utilized wavelet phasor mean fields and wavelet linear models to quantify spatial synchrony for mosquitoes and climate and to calculate the importance of climate in explaining *Cx. tarsalis* synchrony. We also tested whether the strength of spatial synchrony may vary directionally across years. We found significant annual synchrony in *Cx. tarsalis*, and short-term synchrony during a single period in 2018. Mean minimum temperature was a significant predictor of annual *Cx. tarsalis* spatial synchrony, and we found a marginally significant decrease in annual *Cx. tarsalis* synchrony. Significant *Cx. tarsalis* synchrony during 2018 coincided with an anomalous increase in precipitation. This work provides a valuable step toward understanding broadscale synchrony in a WNV vector.

## Introduction

Mosquito borne pathogens are a major threat to human and veterinary health^1^. These pathogens are nested within dynamic systems that include one or more arthropod vectors and vertebrate hosts that must interact in space and time for transmission to be maintained in the natural environment^2^. Several factors contribute to the distribution and magnitude of transmission hazard in an area, including intrinsic population dynamics of vectors and hosts, and extrinsic environmental conditions that can affect the timing, abundances, and distributions of disease system components^3^. Mosquito vectors are one component in these systems, and like all insects, are ectotherms, with their distributions and abundances closely linked to exogenous environmental variables^4^. Because of these linkages and the role of mosquito vectors in pathogen transmission, environmental drivers of mosquito population dynamics are often the focus of investigations attempting to understand outbreaks or epizootic events^5^. While informative, the majority of these studies have focused on environmental effects on local-scale variation in abundances and do not take into account how mosquito populations fluctuate together over space and time at broader scales and extents, despite the potential for synchronous or asynchronous population dynamics in different geographic locations to affect distributions of pathogen transmission^6–8^.

Spatial synchrony, i.e. similarities in temporal fluctuations of populations occurring across geographically distinct locations^9^, is an ecological phenomenon observed across multiple taxa and across local to broad geographic scales. In insects, spatial synchrony has most often been studied in groups that have periodic “outbreaks”, e.g. spongy moths or larch budmoths, and crop pests^10–15^. However, spatial synchrony has been less studied in mosquito vectors of human and veterinary pathogens^16^. Mechanistic drivers of spatial synchrony consist of dispersal between populations, exogenous environmental conditions (through a process called the Moran effect), and trophic interactions of the focal species with another species that exhibits synchrony or that is very mobile^9^. Given known correlations between the environment, particularly temperature and precipitation, and mosquito population dynamics^17,18^, mosquito species are strong candidates for investigations of environmental causes of spatial synchrony across different spatiotemporal scales^19^.

One challenge to previous studies of spatial synchrony in mosquito populations is the need for relatively long and consistent collection records across disparate geographic locations. The result is that the majority of prior population studies occur over small geographic areas or elevational gradients^8,16,19–21^. However, broadscale spatial synchrony is not uncommon in other insect species. Sheppard et al.^13^ found broadscale spatial synchrony in the timing of adult flight onset of aphid species driven by climate conditions at multiple timescales, and Haynes and Walter^12^ highlight how understanding spatial synchrony in insects across scales can be useful to informing pest management decisions. In addition, vector borne disease dynamics in multiple systems have been linked to large-scale climate conditions, including anomalous or unusual conditions, for example: Rift Valley fever virus and heavy precipitation resulting from El Niño in East Africa^22^; broadscale chikungunya incidence associated with unusually dry conditions^23^; and dengue incidence and El Niño-associated dry conditions or elevated temperatures in Thailand^24,25^, among others^26^. Needed are studies to determine if mosquito vector abundances show strong spatially synchronous dynamics at broader extents and timescales and whether their dynamics are associated with climate conditions which often also vary over large spatial extents.

The National Ecological Observatory Network (NEON) conducts routine mosquito collections at 47 terrestrial core and terrestrial gradient sites in the United States^27,28^. The sampling design, temporal resolution, and protocols of mosquito collections at NEON sites were designed specifically for the purpose of standardized monitoring of mosquito population abundances, demography, diversity, and phenology, including comparisons among NEON sites at regional to continental scales^28^. This monitoring program uniquely provides a means to generate consistent and continuous time series collections to better understand mosquito population dynamics across broad geographic scales. Here, we use this resource to investigate whether significant spatial synchrony exists in temporal population dynamics of the West Nile virus (WNV) vector species *Culex tarsalis* across 90 mosquito traps across 9 widely distributed NEON sites that have the most continuous collection of data. In particular, we quantify effects of exogenous climate variables on spatial synchrony between study locations.

*Culex tarsalis* Coquillett is a key vector of WNV (family *Flaviviridae*, genus *Flavivirus*), the leading cause of mosquito-borne disease in humans in the United States (CDC 2021a). The virus is maintained in the natural environment between mosquito vectors and avian hosts, and “spills over” to humans and other animals (Campbell et al. 2002, Reisen 2013). *Cx. tarsalis* are multivoltine with a wide geographic distribution^29^. The species is considered a particularly important vector of WNV in the midwest and western regions of the U.S. where it is often associated with agricultural irrigation or ditches^30^. Mosquitoes become active in late spring and early summer. In temperate zones, adult females overwinter and have been observed in underground sheltered areas, but they remain active year-round in warmer climates (Walter Reed Biosystematics Unit, 2024).

We hypothesized that we may find significant spatial synchrony in *Cx. tarsalis* abundances at multiple temporal scales, including an annual effect. We also expected that average minimum or maximum temperature, humidity values, and precipitation may contribute to annual spatial synchrony across the study period. We tested this directly to determine which climate factors are most important. We also tested if unusual or extreme weather events, such as widespread heavy precipitation, which occurred in Summer 2018, contribute to significant spatial synchrony at shorter timescales. Temperature is a well-known driver of insect population dynamics and has been shown to affect *Cx. tarsalis* mosquito development timing^31^, while precipitation is critical in generating aquatic habitats required for multiple mosquito life stages^4^. Humidity can affect mosquito hydroregulation, impacting activity and survival^32^. Investigating the population dynamics of vector species and how they fluctuate together across large spatial scales has the potential to provide useful knowledge about risks mosquitoes pose, if outbreaks are predictable, and how those risks can be mitigated.

## Results

**Figure 1:**
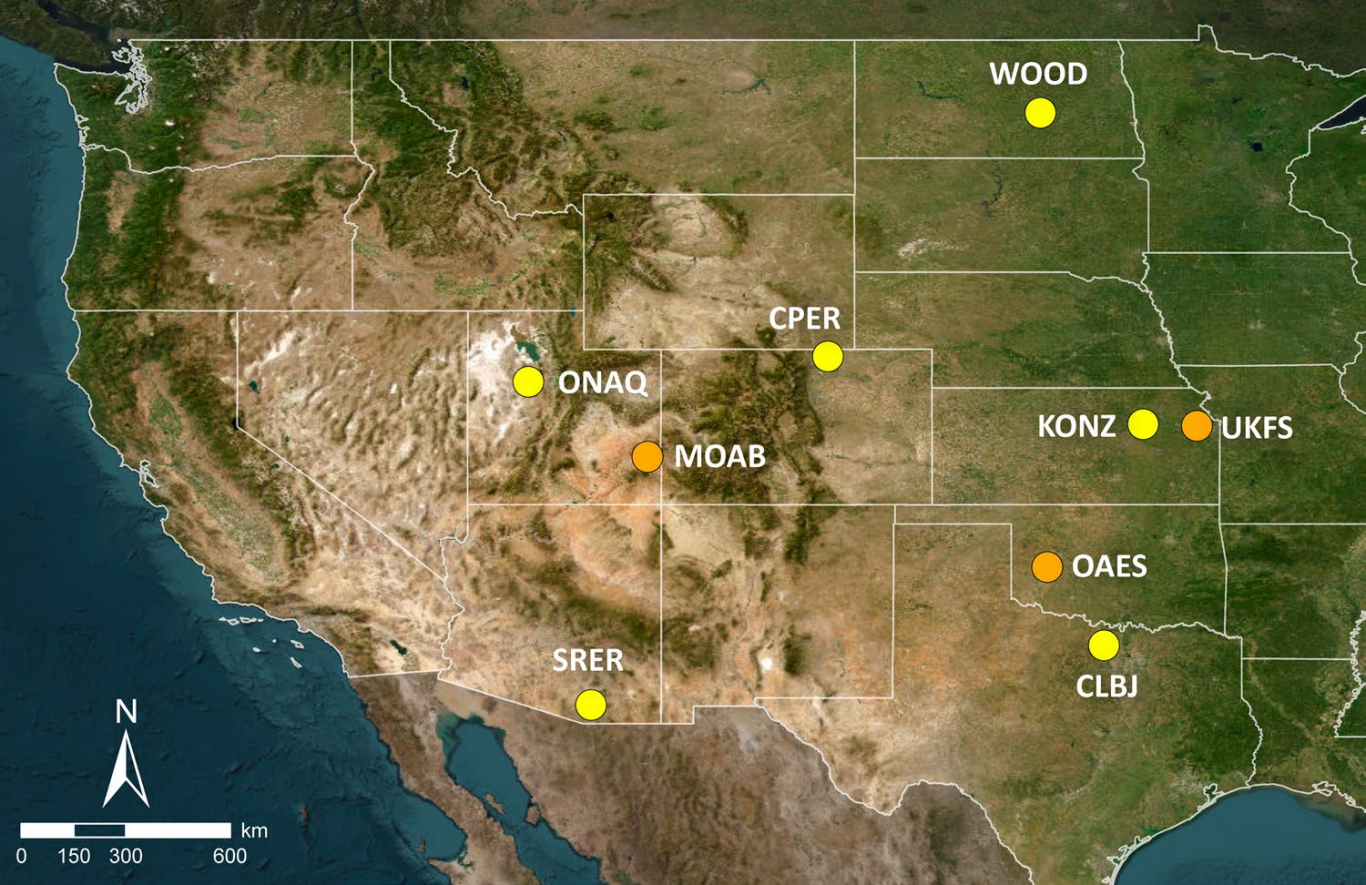
Map of NEON sites included in analyses. Abbreviations for sites follow NEON conventions. Yellow circles indicate terrestrial core sites and orange circles indicate terrestrial gradient sites. Background aerial map Earth Start Geographics SIO, Microsoft Corporation^©^ 2024, available through Bing Maps and accessed through QGIS v 3.16.16.

**Figure 2:**
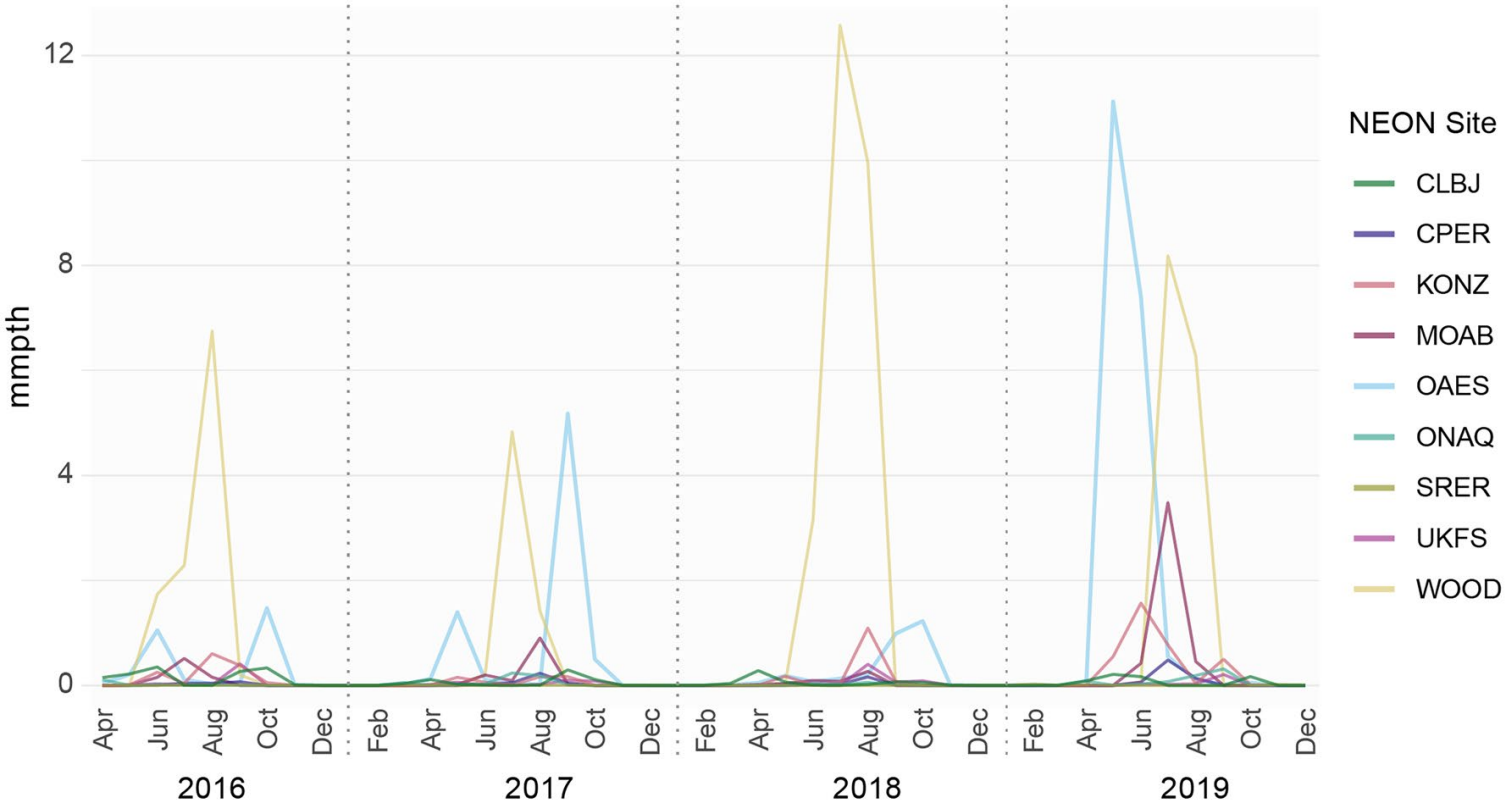
Time series of monthly mean number of *Cx. tarsalis* per trap hour for each NEON site.

Following QA/QC assembled monthly time series of estimated abundances of *Cx. tarsalis* mosquitoes we included nine NEON stations in our analyses over a 45 month period from 2016 to 2019, and associated gridMET climate data (Figs. 1 and 2). We utilized wavelet phasor mean fields (wpmf) to quantify spatial synchrony at multiple timescales for mosquitoes and climate (see Methods for full details of cleaning and analysis approach).

**Figure 3:**
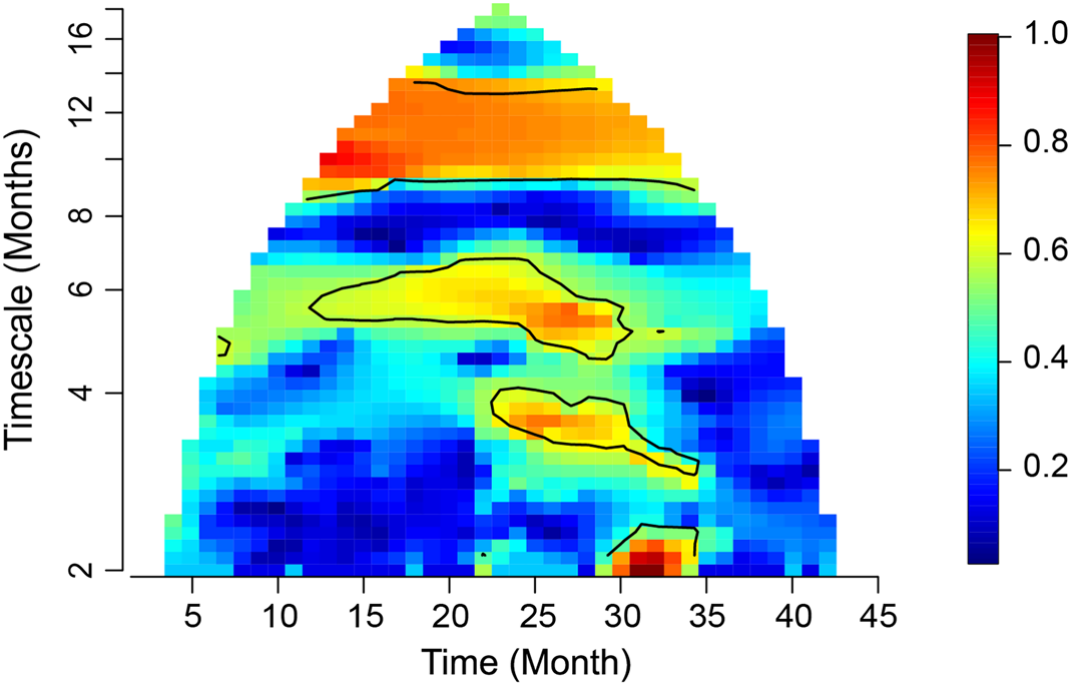
Wavelet phasor mean field plot for *Cx. tarsalis* abundances. The study period is represented on the x-axis, and the timescale of spatial synchrony is on the y-axis. Color corresponds to the strength of phase synchrony in the data at each time and timescale; so areas in red indicate stronger synchrony. Contour lines indicate statistical significance at 95% confidence level.

Results of wavelet phasor mean field (wpmf) analyses indicated significant spatial synchrony in *Cx. tarsalis* abundances at an annual timescale across the study period; and during a short-term, isolated event at the 2 to 3 month timescale band between August and September 2018 (Fig. 3). Although the wpmf output also showed seasonal spatial synchrony (i.e. ~ every six months), this result was likely a harmonic of the annual timescale synchrony.

The plot of the wpmf for *Cx. tarsalis* showed a potential decrease in the strength of annual-timescale spatial synchrony across the study period. Comparing this possibility to an appropriate null hypothesis based on a bootstrapping technique (Methods) indicated a marginally significant (e.g. p-value between 0.05 and 0.10) decrease in the slope of *Cx. tarsalis* synchrony at the averaged 10 to 14 month timescale (*p*-value = 0.096), but when considering only the 12 month timescale on its own (*p*-value = 0.242). Wavelet methods are well known to commit “leakage,” whereby periodic variational content in time series at a given timescale is detected also at a range similar timescales. This is because of the finite length of time series, and tradeoffs between a wavelet’s abilities to localize a phenomenon in time and timescale space. For reasons of leakage, the results here using the 10 to 14 month band are probably more indicative of annual-timescale behavior than are the results using solely the 12-month timescale. Analogous slopes for climate variables at the same timescales were not significant (Supplementary Table 1), i.e., there was no evidence for changes in the strength of synchrony of climate variables.

**Figure 4:**
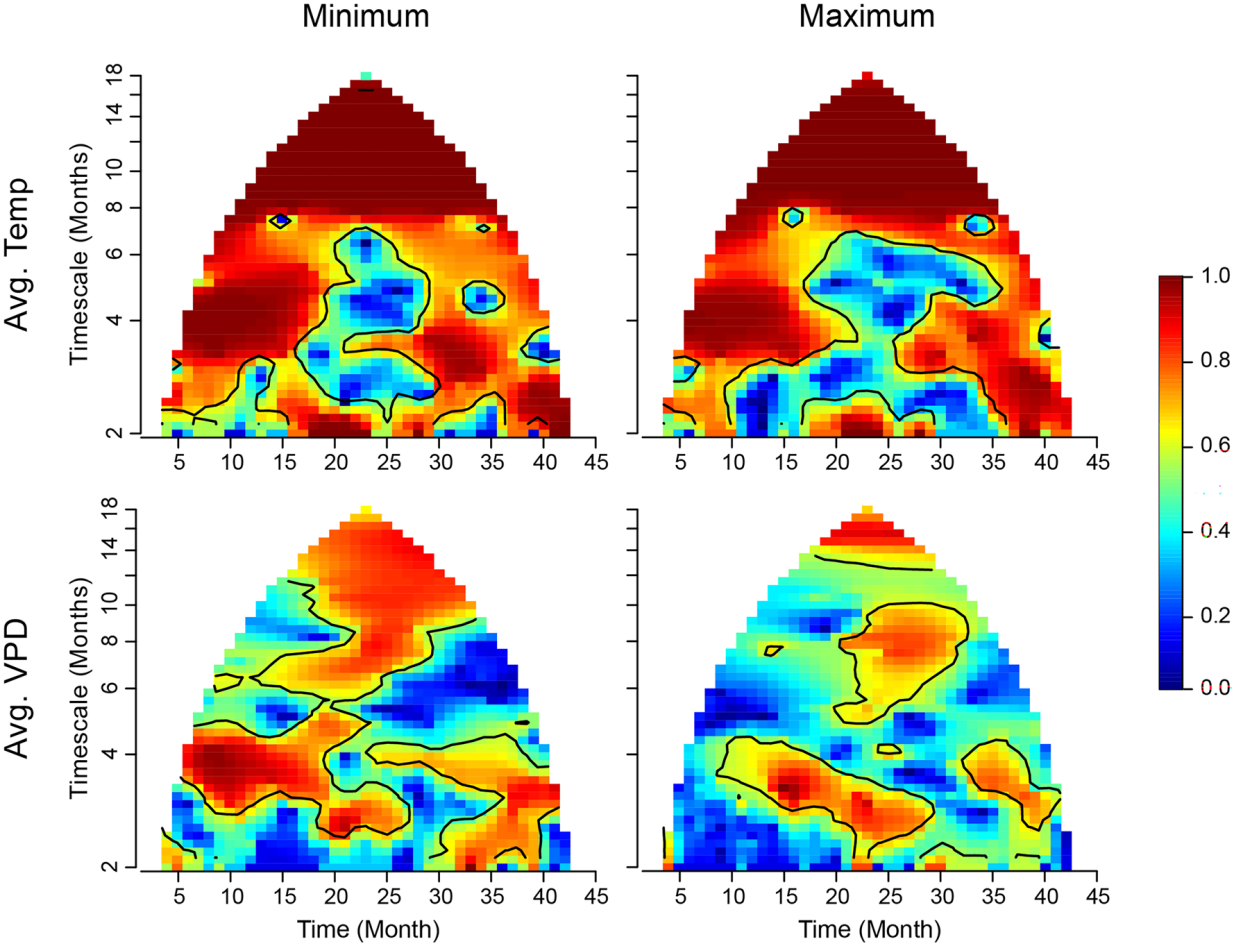
Wavelet phasor mean field (wpmf) plots for average minimum and maximum temperature and average minimum and maximum vapor pressure deficit values.

**Figure 5:**
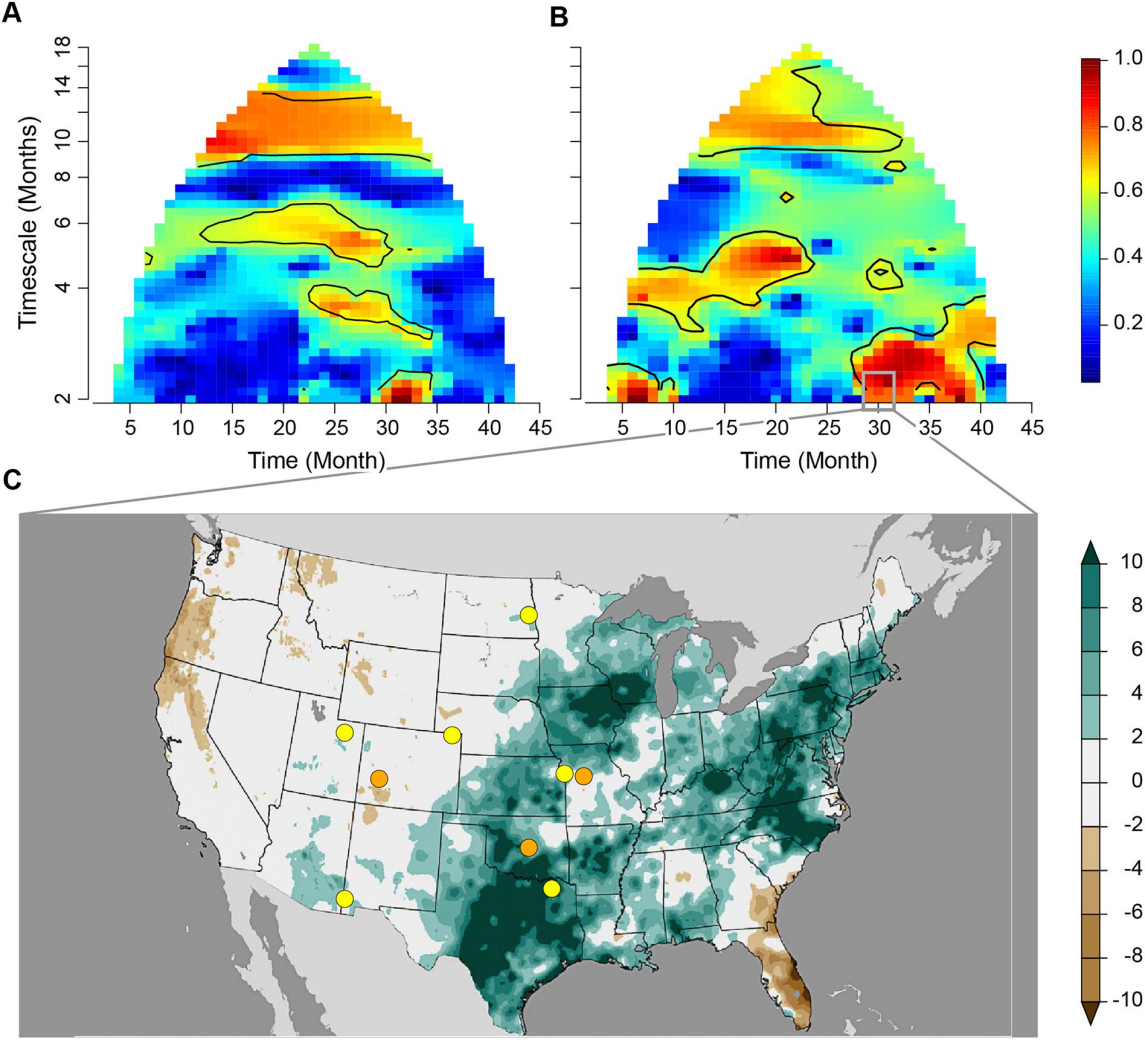
(**A**,**B**) Wavelet phasor mean field plots for *Cx. tarsalis* monthly mean number of mosquitoes per trap hour (reproducing Fig. 3) and monthly cumulative precipitation for the same time periods and locations. Time on the x-axis represents the monthly time series from April 1, 2016 to December 31, 2019. Timescale on the y-axis represents the timescale, or period. Red values enclosed in black lines indicate significant spatial synchrony. C Precipitation anomaly map for August to October 2018 adapted from NOAA (https://www.ncei.noaa.gov/access/monitoring/us-maps). Units are in inches representing the departure from average precipitation over the 1901 to 2000 time period. Areas in green represent greater than average precipitation and areas in brown represent less than average precipitation.

The wpmf plots for four out of five environmental variables showed patterns of significant spatial synchrony at similar timescales and time periods to *Cx. tarsalis* (Figs. 4 and 5B; Fig. 5A reproduces Fig. 3 for visual comparison). As expected, cumulative precipitation and temperature variables showed significant annual synchrony, with both mean minimum and maximum temperature demonstrating the strongest synchrony across the entire study period. Average minimum and maximum mean VPD showed significant synchrony across small portions of the study period at the annual timescale. Cumulative precipitation also showed significant spatial synchrony between August and September 2018 (Fig. 5B).

Results from wavelet linear model tests showed significant effects of synchrony in mean minimum temperature on synchrony in *Cx. tarsalis* at the 10 to 14 month time scale (*p*-value = 0.049). Mean minimum vapor pressure deficit index, i.e., greater humidity, was marginally significant (*p*-value = 0.090), and mean maximum temperature (*p*-value = 0.125), mean maximum vapor pressure deficit index (*p*-value = 0.472), and cumulative precipitation (*p*-value = 0.725) were not significant. Mean minimum temperature explained a large portion of the synchrony (99.490%) with low cross-terms (-1.212), and residuals (1.722). A rank plot with corresponding band tests for mean minimum temperature and wavelet linear model results for mean minimum temperature are available in Supplementary Fig. 1 and Supplementary Table 2.

Although spatial synchrony in cumulative precipitation was nominally significant between August and September 2018 at the 2 to 3 month timescale, the wavelet linear model test was not significant for 2 to 3 month timescales. This result was expected, given that synchrony in *Cx. tarsalis* was isolated to a short-term event and periodicity was not present at the 2 to 3 month timescale; wavelet linear models test for consistency of phase relationships between response and putative predictor variables over the duration of the time series and across sampling locations, and apparently what happened on 2 to 3 month timescales in our *Cx. tarsalis* data was instead a one-off event. However, observations of precipitation values coinciding with this time period revealed that anomalously high precipitation occurred across several study sites, and a visual inspection of estimated abundance values across NEON sites showed an increase in *Cx. tarsalis* during this time period across multiple sites (Fig. 5C, see Data Availability).

## Discussion

This study provides some evidence of broad scale spatial synchrony in *Cx. tarsalis* abundances, and results demonstrate that *Cx. tarsalis* populations can fluctuate together at an annual timescale and during short-term, isolated events. This work highlights the need for continued investigation to understand spatiotemporal and synchronous dynamics of mosquito vectors and their drivers. These dynamics, which our results suggest may be predictable, may help in understanding broader WNV system dynamics.

Our finding of significant spatial synchrony at an annual timescale was expected given the seasonal dynamics of mosquitoes and insects in general^33,34^. Specifically, we found that mean minimum temperatures were a significant predictor of synchrony in *Cx. tarsalis* abundances at an annual timescale, i.e., mosquito synchrony on annual timescales was statistically attributable to synchrony in mean minimum temperatures on the same timescale range. Temperatures are known to produce synchronizing effects on insects through multiple mechanisms, including a cessation of activity during winter diapause, temperature dependent development where minimum temperature thresholds must be met for development to occur at different life stages, and temperature dependent mortality at different life stages^35^. Each of these factors or combinations of these factors could have synchronizing effects on *Cx. tarsalis* activity and abundances, particularly across a large geographic area that includes temperate environments where populations undergo winter diapause^36,37^. In overwintering mosquitoes, termination of diapause begins with extended photoperiod and warming temperatures, which is then followed by a post-diapause development period that can result in synchronous spring activity once a minimum temperature threshold is reached^38^. In addition, minimum temperatures could produce synchronous abundances through mortality if minimum temperatures are too cold.

We found that the strength of annual *Cx. tarsalis* synchrony showed a marginally significant decrease across the study period, but we did not observe a significant decrease in the strength of annual synchrony of minimum temperature across the study period. According to the wavelet Moran theorem of^13^, two factors contribute to the strength of synchrony induced in a population variable by a synchronous environmental variable: the strength of synchrony of the environmental variable; and the strength/consistency of the relationship between the population variable and the environmental variable. Thus, annual timescale *Cx. tarsalis* synchrony could have been caused by temperature synchrony, and could have declined over our study period even while temperature synchrony held steady, if the influence of temperature on mosquito annual dynamics became less pronounced over the study period. This could have happened, for instance, if an overall warming trend in minimum temperatures interacted with threshold dependencies in mosquito life history processes so that thresholds were less frequently a limiting factor in the growth of some populations^38,39^. In general, warmer diapause temperature has been found to desynchronize eclosion in the green-veined white *Pieris napi* Linnaeus (Lepidoptera:Pieridae) butterflies^40^. Other factors could also contribute to a decrease in annual synchrony in *Cx. tarsalis* even while temperature synchrony remains strong: increased local stochasticity in population dynamics, altered biotic interactions, or increasing variation in asynchronous regional environmental conditions that drive local *Cx. tarsalis* dynamics could all result in reduced annual spatial synchrony across the study period.

An outstanding question that can only be addressed through continued long-term mosquito collections is whether the marginally significant result of a decrease in annual spatial synchrony in *Cx. tarsalis* observed here is part of a longer-term, consistent trend, or instead is part of a periodic phenomenon, so that the decline in synchrony will be reversed in due course^12^ provide a summary of insect populations where longer-term shifts in the strength of spatial synchrony have been observed, and highlight the need for longer-term monitoring to understand synchronous population dynamics and the effects of global climate change on insects. Reuman et al. (In Review) point out that, even for systems where time series length is long (those authors consider some time series which are almost 2000 years long), the evidence suggests that yet longer time series can reveal even longer-timescale synchrony. For mosquito vector species, understanding these patterns is particularly relevant because the strength of spatial synchrony across multiple timescales may be informative for understanding and predicting for the timing and abundance of mosquitoes, which relates through a set of other complex processes to transmission risk.

In addition to significant annual synchrony, we found evidence of significant synchrony during a single, short-term event that occurred between August and September 2018. These results demonstrate that spatial synchrony in *Cx. tarsalis* is not confined to annual cycles alone and emphasizes the need to consider how short durations or individual events can lead to one-off, spatially synchronous elevations in abundances. The timing of this event is relevant because it occurred during the peak WNV transmission season and during a year with broadscale spillover^41^. Here we show that precipitation between August and October 2018 was unusually high, with record breaking values occurring across several sites in and around our study area (Fig. 5C) (https://www.ncei.noaa.gov/access/monitoring/us-maps).

Although synchrony in precipitation was not a significant predictor of synchrony of *Cx. tarsalis* during this event, this result was not unexpected given that spatial wavelet analyses focus on periodicity, and single events that are short in duration may not trigger significant wavelet coherence in wavelet linear models. However, observations of strong spatial synchrony in *Cx. tarsalis* and knowledge of anomalous precipitation during the same time period warrants further investigation. Understanding not only interannual patterns but effects of the timing and distribution of intra-annual, widespread, anomalous weather events on mosquito population synchrony may reveal environmental drivers that are precursors to potential elevated vector borne disease transmission hazard. Investigation of such isolated events should probably proceed via statistical methods other than the wavelet approaches we have used here.

We close by noting some limitations of this work and next steps. First, while the geographic coverage of the NEON sites spanned a broad area, our study was limited to 90 mosquito traps across 9 locations owing to sampling coverage and distribution of *Cx. tarsalis *mosquitoes. Second, we lack the longer time series needed to determine synchrony on timescales longer than about 16 to 18 months. Third, short time series limit statistical power to detect environmental drivers of synchrony, especially on longer timescales, which is demonstrated in our finding of a marginally significant decrease in the strength of spatial synchrony in *Cx. tarsalis* abundances over our study period. To establish significance of a relationship between mosquitos and temperature, the wavelet methods we used looked for consistency, across both space and time, in phase differences between annual-timescale fluctuations in *Cx. tarsalis *and temperature. But, on annual timescales, only a few oscillations of both variables occur during the duration of our data, limiting the potential of our methods to detect consistent phase relationships. Longer datasets would mitigate this problem. In addition, although we conducted a broadscale analysis of *Cx. tarsalis* mosquitoes, the distribution of the NEON sites encompasses a relatively small portion of their geographic range, and our findings are specific to the study region. Although, evidence indicates mosquito-mediated dispersal of West Nile virus in the western United States^42^, we consider it unlikely that dispersal is a driver of synchrony on the spatial scales we examine because of the large geographic distance between NEON sites in our analyses.

This work sets the stage for integrating longer time series over more sites, including in regions outside the United States where *Cx. tarsalis* are abundant and to other WNV mosquito vectors. Longer-term time series of mosquito collections from monitoring programs including NEON or from mosquito trap collections conducted by mosquito control programs may provide the opportunity to investigate more robustly environmental correlations with unusual events and to identify common precursors, such as periodic ENSO oscillations, to synchronous population fluctuations.

Despite limitations, this work provides a first step toward understanding broad scale population synchrony in a key WNV mosquito vector. Our results highlight the need for additional investigations into effects of synchronous population dynamics on disease system dynamics during normal and unusual environmental conditions, over multiple timescales and different geographic areas. Future investigations will benefit from a systems approach that includes not only environmental correlates but other components of the WNV disease system, including avian host populations, migration phenology, and detected spillover events. Understanding the timing and distribution of inter- and intra-seasonal synchronous mosquito vector population dynamics across geographic scales may provide needed insight and a piece of the puzzle toward overcoming outstanding challenges in predicting the magnitude and extent of WNV transmission. Overcoming these challenges can ultimately help better inform prevention and control agencies.

## Materials and methods

National Ecological Observatory Network (NEON) routine mosquito collections (Product: DP1.10043.001) between April 1, 2016 and December 31, 2019 were used in this analysis^43^. Mosquitoes at NEON sites are collected using CO_2_ baited Centers for Disease Control (CDC) light traps, and within each NEON site, mosquitoes are typically collected at ten trap locations, referred to as plots. Traps are set at a minimum distance of 310 m, and here, the maximum distance between 2 traps occurred at one NEON site (SRER) with a distance of ~ 14.36 km. Terrestrial core sites collect mosquitoes every two weeks during the field season and then sample three trap locations weekly during the off season^27,28^. Following three consecutive trapping events with zero mosquitoes collected, terrestrial core sites change their sampling scheme to the off season protocol where traps are set weekly at three plots. This increase in temporal sampling at a reduced number of sites is designed to capture the return of adult mosquito flight activity following winter conditions. Terrestrial gradient sites collect mosquitoes every four weeks during the field season, cease collections when the terrestrial core site within their domain changes to the off season protocol, and then resumes collections when mosquito activity begins at the terrestrial core site^27,28^. Here, we conducted our analyses at a monthly temporal resolution and used the following steps to assemble time series of *Cx. tarsalsis* abundances for analyses.

First, we reduced site locations to those that had collected at least one *Cx. tarsalis* mosquito between 2016 and December 2019 for a total of 26 candidate sites. Next, we performed QA/QC on individual sampling records to ensure that trap issues did not compromise sampling. We considered a trap collection compromised if any event occurred that could affect the mosquito counts collected or recorded. These events included interference with CO_2_ sublimation because of blockage, traps tipped over and on the ground, holes in catch cups, ants in catch cups, samples frozen and irretrievable from the sides of catch cups, damaged samples, or lost and discarded samples. In each of these cases, the number of mosquitoes or the magnitude of damage to the sample could not be assessed, introducing an unknown level of uncertainty to abundances. Individual counts recorded in NEON data reflect the number of mosquitoes per species identified out of a subsample of the total trap collection. In order to estimate the *Cx. tarsalis* abundance for each record, the individual count is divided by the proportion of the total trap collection identified. We calculated the estimated count and then created a monthly template beginning April 1, 2016 and ending December 31, 2019. If a trap collection did not include *Cx. tarsalis*, we entered a zero for the count, and if the trap was not set during the month, we entered NA.

Next, we estimated trapping effort and calculated *Cx. tarsalis* counts per trap hour per month across the 10 locations within each of the 26 NEON candidate sites for each month of the study period, resulting in one value per site per month. Following this step, we checked sample coverage across the study period for each NEON site and reduced the analysis to nine sites with consistent sampling during active mosquito time periods. These sites consisted of six terrestrial core sites and three terrestrial gradient stations, and they are predominantly located in the interior West and Great Plains region (Fig. 1). Because the spatial wavelet analysis software we will apply (see below) requires continuous time series, we replaced NA values with zeros during winter and early spring months for temperate sites when collections did not occur, using the assumption supported in the literature^36,37,44^, that *Cx. tarsalis* mosquitoes were not active during winter time periods. We note that cessation of sampling during winter must be preceded with 0 counts in abundance at the terrestrial core site within the NEON domain, providing a strong basis for making the assumption that mosquitoes are not active. All sites with mean number of mosquitoes per trap hour per month are available in supplementary materials, including NA values when sampling did not occur (see Data Availability).

After assembling and cleaning site-level data, we then calculated the centroid of the ten mosquito trap locations within each NEON site for each of the nine sites to obtain a single geographic point reference for environmental data preparation. GridMET daily cumulative precipitation, minimum and maximum temperature, and minimum and maximum mean vapor pressure deficit index (a measure of humidity) at a 4 km spatial resolution were downloaded for each NEON site between April 1, 2016 and December 31, 2019 using the ‘climateR’ package^45,46^. Daily data was binned by month, and we calculated total cumulative precipitation for each month, and average values for all other variables (e.g., minimum and maximum temperature and relative humidity) using functions available in the ‘dplyr’ package in R^47^.

Once monthly abundance and climate data were assembled, we used the ‘wsyn’ package in R for spatial synchrony analyses^48^. First, we used the ‘cleandat’ function with clev = 3 to individually de-mean, detrend, and standardize the variance of the monthly time series for *Cx. tarsalis* and each environmental variable. Next, we calculated wavelet phasor mean fields (wpmf) to quantify whether significant spatial synchrony occurred at one or more timescales for monthly estimated abundances of *Cx. tarsalis* and each environmental variable. Given a collection of time series measured at the same times (e.g., our *Cx. tarsalis* time series), the wpmf technique provides a plot which displays the strength of phase synchrony in the input time series as a function of time and timescale, with significance contours. Intense colors on the plot indicate strong synchrony at the given time and timescale. This method and a suite of closely related and now well developed methods have been applied numerous times to study synchrony of ecological time series^15,49–56^, and the methods are implemented, open source, in the wsyn package on CRAN^48^. The wsyn package includes a “vignette” which gives a straightforward, operational introduction to the methods implemented therein.

Next, we observed the wpmf plot for *Cx. tarsalis* to determine whether spatial synchrony was significant across one or more timescales. We then fit univariate wavelet linear models using the ‘wlm’ and ‘wlmtest’ functions in wsyn to quantify whether spatial synchrony in the climate variables were significant predictors of spatial synchrony in *Cx. tarsalis* at the same timescales. Wavelet linear models were originally developed by^13,54^, and have now been applied several times in ecology^15,49–53^ to identify environmental causes of synchrony; they are especially useful when causes of synchrony may differ by timescale. These tools can identify not only which environmental drivers likely help cause synchrony on a given timescale band, they can also indicate the fractions of synchrony explained by each driver and by interactions between drivers. Wavelet linear model methods make statements of statistical significance based on resampling/randomization procedures; the number of randomizations in the ‘wlmtest’ function was set to 10,000. The ‘bandtest’ and ‘plotrank’ functions in wsyn were used to quantify significance. If a climate variable was identified as a significant driver of synchrony in *Cx. tarsalis*, the percent synchrony explained by the variable was then obtained from the wlm model, with associated cross-terms and residuals.

After observing the wpmf output for *Cx. tarsalis*, we tested whether a significant decrease in the strength of spatial synchrony occurred across the study period at the annual timescale by generating 10,000 “synchrony preserving surrogates” using the ‘surrog’ function in wsyn, with surrtype = ‘aaft’^54^. Synchrony preserving surrogates are so-called surrogate datasets, i.e., artificial, bootstrapped datasets of the same structure as the original data (same number, length, and sampling frequency of time series), but which have been randomized in such a way that synchrony between the time series is maintained in its strength and timescale structure, but any directional changes through time in synchrony are eliminated. Thus, comparing patterns of change through time in the synchrony of real data against the same patterns computed for the surrogate datasets provides a test of whether synchrony has directionally changed, to a significant extent, against an appropriate null hypothesis.

We calculated the synchrony values for our observed *Cx.tarsalis* data at the 12 month and averaged 10 to 14 month timescales, regressed these quantities against year to obtain two slopes, and calculated the same statistics for each of the 10,000 synchrony-preserving surrogate datasets. We then calculated the proportion of the 10,000 surrogate slopes which were less than the observed slopes to obtain a *p*-value for the test of the null hypothesis that observed decreases in annual-band *Cx.tarsalis* were no more than could have been expected by chance. Significance was measured with an ɑ < 0.05 and we report marginal significance with an ɑ < 0.10. We also tested whether a significant decrease in the strength of annual spatial synchrony was present in the climate variables, using the same methods.

### Supplementary Information

Supplementary Information.

## Data Availability

The data used in this study, including the data file prior to filling NA values, is available through GitHub (https://github.com/Campbell-Lab-FMEL/Culex-tarsalis-synchrony).

## Code availability

All code used to perform analyses and to create figures is available through GitHub (https://github.com/Campbell-Lab-FMEL/Culex-tarsalis-synchrony).
