# Supplementary material for: Broadscale spatial synchrony in a West Nile virus mosquito vector across multiple timescales
Source: Sci Rep. 2024 May 30;14:12479. doi: 10.1038/s41598-024-62384-6 (PMC11139987; doi:10.1038/s41598-024-62384-6)

Supplementary Table 1. Analyses testing for a significant decrease in the strength of spatial synchrony across the study period at the averaged 10 to 14 timescale using 10,000 surrogate slopes

| Variable | Slope | p-value |
| --- | --- | --- |
| Culex tarsalis | -0.008 | 0.096 |
| cumulative precipitation | -0.008 | 0.164 |
| mean maximum VPD | 0.010 | 0.740 |
| mean minimum VPD | 0.021 | 0.837 |
| mean maximum temperature | 0 | 0.421 |
| mean minimum temperature | 0 | 0.484 |

Supplementary Table 2. Results from wavelet analyses at the 10 to 14 month timescale for climate variables with significant and marginally significant wavelet tests.

| Variable | % Synchrony Explained | Crossterms | Residuals | p-value |
| --- | --- | --- | --- | --- |
| mean minimum temperature | 99.490 | -1.212 | 1.722 | 0.049 |
| mean minimum VPD | 42.769 | 35.837 | 21.394 | 0.090 |

Supplementary Figure 1. Rank plot showing the results of the ‘wlmtest’ for mean minimum temperature at the 10 to 14 month timescale.


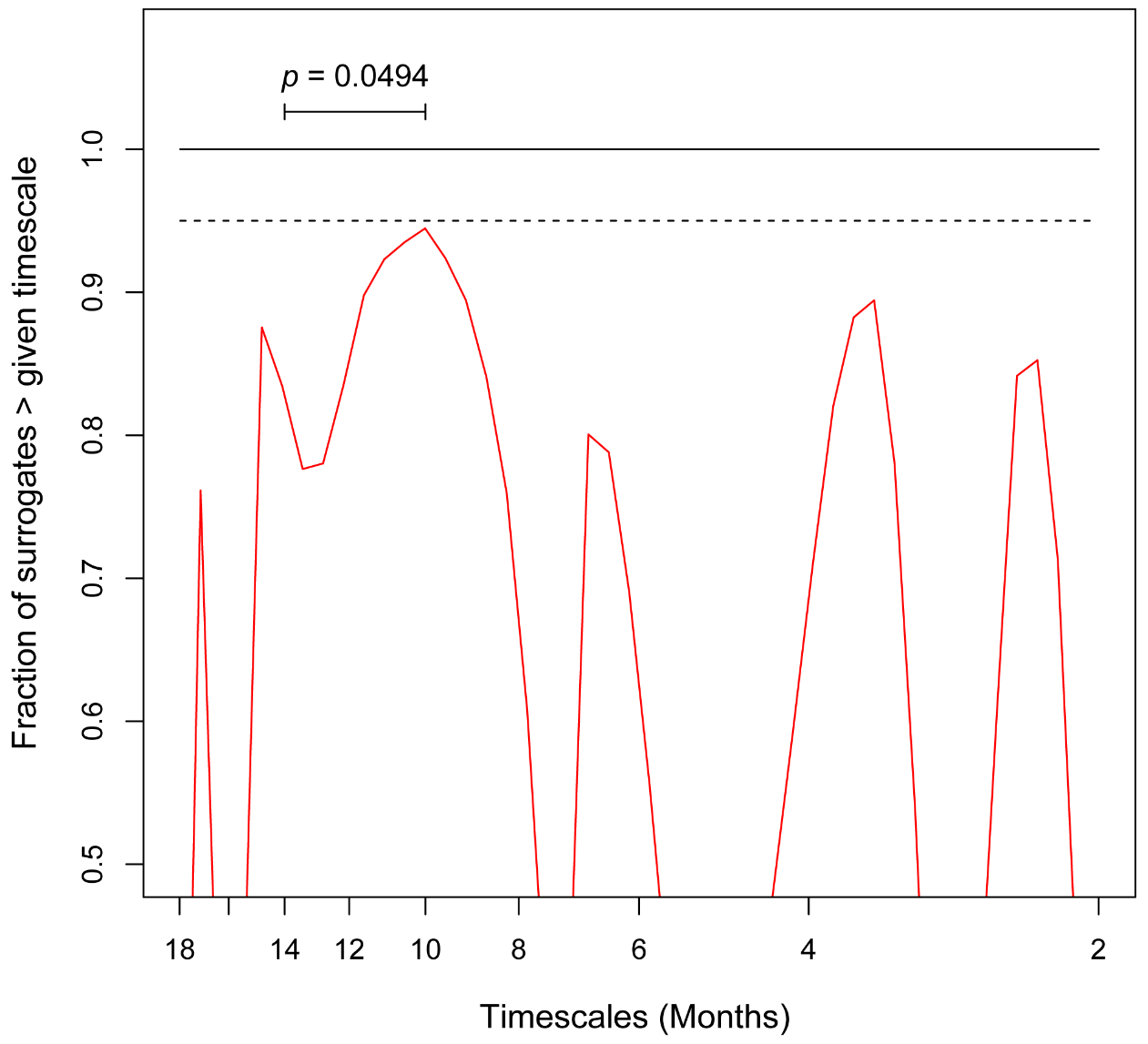

Supplement: Supplementary file 1 — Supplementary Information. [file 41598_2024_62384_MOESM1_ESM.docx]
